# Lung Entrapment Secondary to Loculated Rheumatoid Effusions Treated With Alteplase and Dornase

**DOI:** 10.7759/cureus.66635

**Published:** 2024-08-11

**Authors:** Sagar Kumar, Huma Akta, Davis Weidow, Matthew Clukies, Ji Young Lee

**Affiliations:** 1 Pulmonary and Critical Care, University of South Alabama, Mobile, USA; 2 Internal Medicine, Franklin Primary Health Center, Mobile, USA; 3 Physiology and Cell Biology, Center for Lung Biology, College of Medicine, University of South Alabama, Mobile, USA

**Keywords:** rheumatoid arthritis, chest tube drainage, dornase alfa, alteplase (tpa), loculated pleural effusion, entrapped lung

## Abstract

Rheumatoid arthritis is a multisystemic inflammatory disease that can involve the respiratory system, including the pleural space. Most rheumatoid pleural effusions (PE) are incidentally found and do not require any treatment. Very rarely, however, they can become symptomatic and loculated, leading to lung entrapment or trapped lung. Surgical decortication remains the mainstay of management in such circumstances, although recent studies showed comparable efficacy of intrapleural fibrinolytics (alteplase and dornase alfa) in non-rheumatoid complicated effusions.

We present a case of rheumatoid PE leading to lung entrapment successfully treated with intrapleural fibrinolytics without complications and good clinical status at six-month follow-up.

## Introduction

Rheumatoid arthritis (RA) is a multisystemic inflammatory disease that primarily affects joints but can present with extra-articular pulmonary manifestations, including interstitial lung diseases, small and large airway diseases, lung nodules, and pleural effusions (PE) [1]. Asymptomatic PE in RA are common, but they can be symptomatic in 3-5% of the cases. They tend to be exudative and are associated with high lactate dehydrogenase (LDH), high RA factor titers, low pH, and low glucose, and diagnosis generally requires ruling out other causes of PE, including tuberculosis and malignancy [2]. Asymptomatic RA-associated PE (RPE) can be managed with clinical surveillance, as most effusions spontaneously resolve over time. In a minority of cases, systemic or intrapleural steroids are used to treat underlying inflammation, which usually results in clinical recovery [3]. Very rarely, RPE can loculate and compromise respiration via restrictive physiology that has traditionally been managed with surgical decortication in symptomatic patients [2,4]. Intrapleural fibrinolytics (alteplase and dornase alfa) have been established as a less invasive alternative to decortication surgery in patients with complicated effusions and have been shown to have comparable efficacy [5]. However, its role in noninfectious loculated PE remains largely unexplored.

Herein, we present a case of a female with a history of RA who presented with subacute dyspnea and hypoxemia in the setting of loculated bilateral PE with lung entrapment secondary to rheumatoid pleurisy. She was treated with intrapleural fibrinolytics through a small-bore pigtail chest tube, which resulted in significant clinical and radiographic improvement.

This case report was presented as an abstract presentation at the American Thoracic Society (ATS) International Conference 2024, San Diego [6].

## Case presentation

A 63-year-old Caucasian female was referred to the emergency department (ED) by her primary care physician due to a one-month history of progressively worsening shortness of breath, fatigue, and gradually enlarging PE on outpatient serial chest X-rays. Additional symptoms included a recent recurrence of chronic joint pains and subjective fevers in the setting of a known diagnosis of RA for 24 years for which she was not taking any medications. She was otherwise vitally stable except for mild tachypnea and hypertension, and she required 2 liters of supplemental oxygen to maintain saturations >90%. Pertinent laboratory findings and arterial blood gas on 2 liters of supplemental oxygen are mentioned in Table 1.

**Table 1:** Pertinent laboratory findings and arterial blood gas mg/dL: milligrams per deciliter, mMol/L: millimoles per liter, mcL: microliter, g/dL: grams per deciliter, PCO2: partial pressure of carbon dioxide, PaO2: partial pressure of oxygen, mmHg: millimeters of mercury

Lab name	Lab value	Reference range
Serum creatinine	1.82 mg/dL	0.53-1.02 mg/dl
Serum albumin	2.2 g/dL	3.4-5.4 g/dL
WBC	8.4 x 10(3)/mcL	4.0 x 10(3)-11.0 x 10(3)/mcL
Hemoglobin	8.7 g/dL	12.0-15.0 g/dl
Platelet count	540 x 10(3)/mcL	150-450 x 10(3)/mcL
PH	7.37	7.35-7.45
PCO2	50 mmHg	35-45 mmHg
PaO2	67 mmHg	>80 mmHg

**Figure 1:**
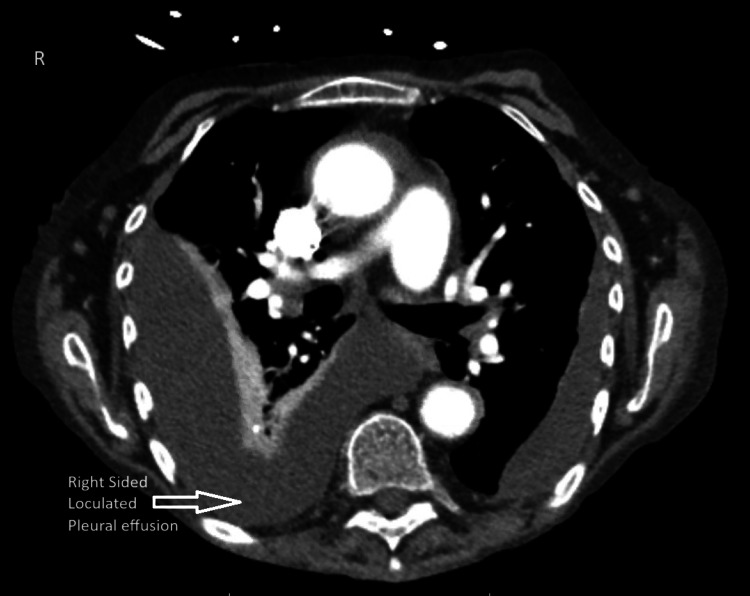
Axial view of CT scan chest showing large right-sided loculated effusion and medium to large left-sided effusion CT: computed tomography

The chest X-ray was concerning for bilaterally loculated (right greater than left) effusions that were confirmed on a CT scan of the chest (Figure 1). A transthoracic echo showed normal heart function.

**Figure 2:**
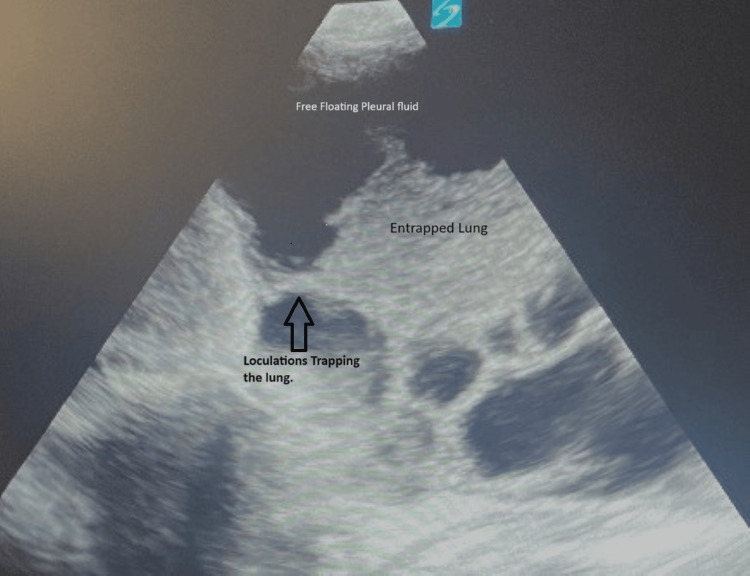
Right-sided lung US showing loculated effusions and multiple septations US: ultrasound

**Figure 3:**
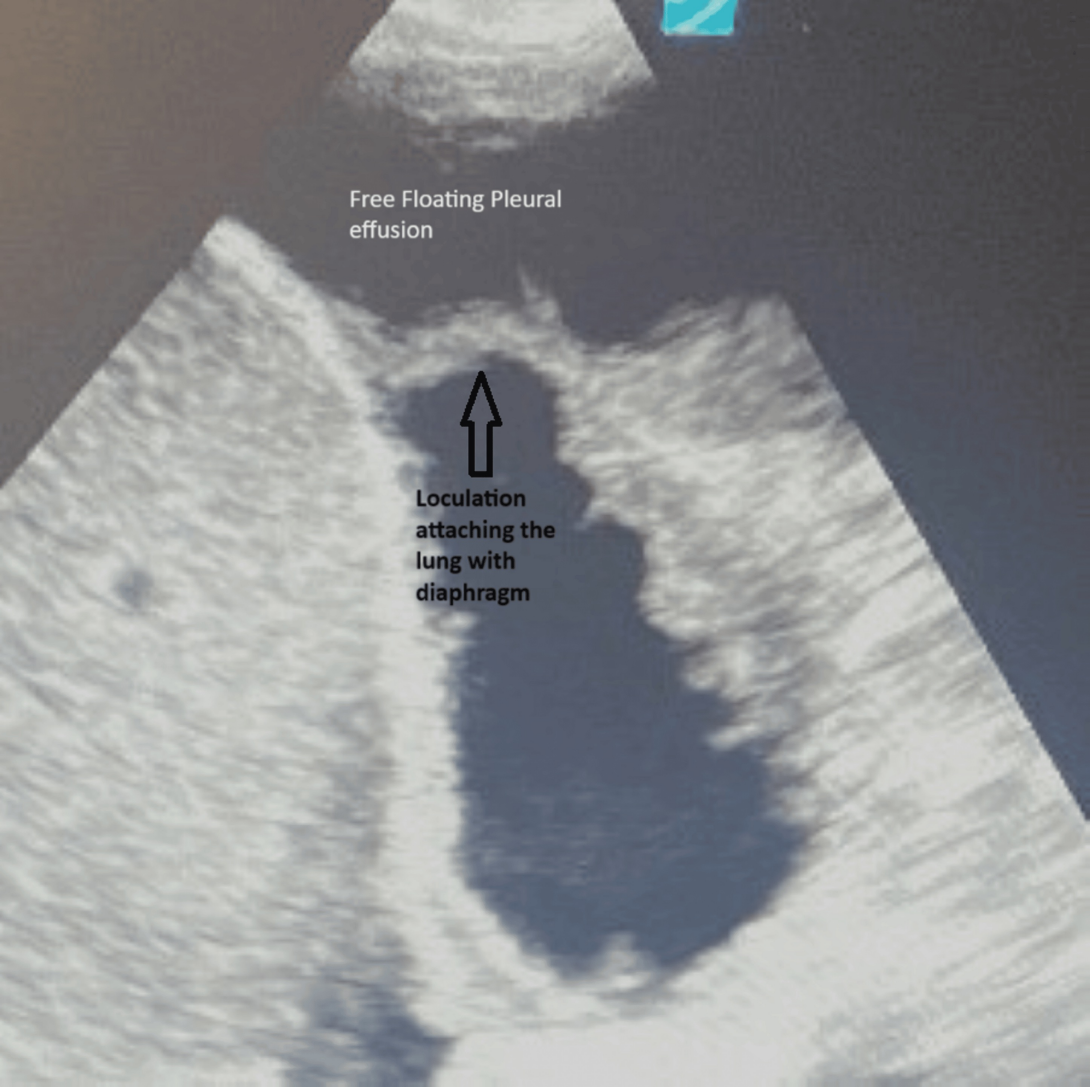
Left-sided lung US showing a fibrin thread (loculations) attached to the diaphragm US: ultrasound

Thoracic ultrasound (US) revealed severely loculated right-sided effusions with some free-flowing fluid and mildly loculated left-sided effusions (Figures 2-3).

Thoracentesis drained 700 cc of yellow, cloudy exudative effusion but had to stop due to severe ipsilateral chest pain with suctioning, raising concern for an entrapped lung. A postprocedural X-ray confirmed inadequate expansion and the absence of pneumothorax. Pleural fluid analysis revealed an exudative effusion with very low glucose, low pH, high LDH, and a high WBC count with lymphocytic predominance. Gram stain, culture, acid-fast bacilli (AFB) polymerase chain reaction (PCR) and culture, fungal cultures, and malignant cells were negative-detailed analyses mentioned in Table 2 as thoracentesis 1.

**Table 2:** Pleural fluid studies from first and second thoracentesis LDH: lactate dehydrogenase, WBC: white blood cells, ADA: adenosine deaminase, AFB: acid-fast bacilli, RA: rheumatoid arthritis, PCR: polymerase chain reaction, F/u: follow-up, m: mili, d: deci, mc: micro, g: gram, U: units, L: liter

Study	Thoracentesis 1	Thoracentesis 2 (2 days later)
PH	7.28	7.13
Glucose	3 mg/dL	4 mg/dL
LDH	650 unit/L	1,804 unit/L
Protein	4.1 g/dL	3.7 g/dL
WBC count	381 cells/mcL	2000 cells/mL
Lymphocytes %	49	4
Neutrophil %	29	95
Eosinophils%	0	0
ADA	48 U/L	51 U/L
Cytology	No anaplastic cells	No anaplastic cells
Gram stain and culture	Negative	Negative
AFB stains, AFB PCR, AFB, and fungal culture (8 weeks F/u)	Negative	Negative
RA factor	Not checked	803 IU/mL

The patient was monitored for two days while an acute bacterial infection was ruled out, and she continued to be symptomatic and hypoxemic. In the setting of a high inflammatory state as reflected by a high serum C-reactive protein of 25 milligrams/deciliters and an erythrocyte sedimentation rate of 130 mm/hour, RA factor 1: 1024 units, anti-cyclic citrullinated peptide: 215 units, active arthritis symptoms, typical pleural fluid analysis without atypical cells x 2, negative QuantiFERON and AFB PCR, and medication noncompliance, this was thought to be RPE. Treatment options were discussed with a multidisciplinary team and the patient, and the decision was to place a small bore (14 French Wayne Pigtail catheter) in the chest tube for treatment with alteplase and dornase.

Two days later, at the time of chest tube placement, repeat thoracentesis showed worsening chemistry findings, including lower pH and glucose, higher LDH, a high pleural RA factor, and very high white blood cell counts with neutrophil predominance. AFB and fungal cultures (followed up for eight weeks) remained negative, and cytology was unremarkable. Further details are shown in Table 2 as thoracentesis 2. Despite empyematous-appearing pleural fluid findings, antibiotics were held as the patient did not show any clinical symptoms or laboratory findings of infection. The patient was treated with intrapleural administration of 10 mg of alteplase mixed with normal saline and 5 mg of dornase alfa mixed with water every 12 hours for three days. Treatment with fibrinolytics resulted in significant drainage of serosanguineous fluid over the next three days, resulting in dramatic symptomatic and radiographic improvement. The patient was discharged on low-dose prednisone. At a six-month follow-up at the rheumatology office, she was asymptomatic from a respiratory standpoint.

## Discussion

Rheumatoid pleurisy can manifest with a broad spectrum of pleural pathologies. Typically, it presents with asymptomatic, small exudative effusions that often do not necessitate treatment. However, in rare instances, it may lead to complicated effusions such as large exudative sterile empyematous rheumatoid effusions, cholesterol (chyliform) effusions, empyema, hydropneumothorax, or pyopneumothorax, which may require pharmacological or invasive interventions [2,7]. Diagnosis generally requires pleural fluid analysis showing low glucose, low pH, high LDH, and high WBC count. Neutrophilic predominance typically occurs initially, followed by lymphocytic predominance [3]. Interestingly, our patient initially exhibited lymphocytic predominance, followed by neutrophilic predominance upon repeat thoracentesis on the same side. This unusual trend could be attributed to fluid collection from different pockets due to loculations or the newer effusion resulting from ongoing inflammation, as reflected by systemic symptoms and elevated inflammatory markers. The possibility of a new pleural infection after previous thoracentesis or the risk of spontaneous infection in RPE was also considered but was unlikely. There was no fever or leukocytosis, and pleural fluid gram stains and cultures were negative, and the patient clinically improved without antibiotics. It is important to rule out malignancy and tuberculosis, as they may have similar features in pleural fluid analysis. If the diagnosis remains unclear, a thoracoscopic pleural biopsy may be warranted that may show the replacement of normal parietal mesothelial cells with inflammatory cells, tadpole cells, and multinucleated giant cells [8]. We made the diagnosis based on classic pleural fluid chemistry, a very high RA titer in the pleural fluid, multiple negative cytology, and negative cultures and stains for AFB and fungi.

Recommendations for RPE treatment are mostly based on case reports and series. In many cases, RPE resolves spontaneously or requires only intermittent therapeutic thoracentesis for symptom relief [9,10]. Nonetheless, some studies advocate for aggressive treatment approaches including systemic steroids, intrapleural steroids, or other immunosuppressives due to the potential progression of RPE leading to pleural thickening, lung entrapment, trapped lung, or fibrothorax, which may necessitate surgical interventions including decortication and/or pleurectomy [2,4,11,12]. The use of less invasive interventions such as intrapleural fibrinolytics has been increasing in loculated parapneumonic effusions and empyema over the past decade, and recent studies showed their comparable efficacy and better quality of life index compared to surgery [5,13,14]. However, data on treating severely loculated RPE with intrapleural fibrinolytics is very scarce, and to the best of our knowledge, there is only one abstract published regarding the management of loculated RPE with fibrinolytics [15].

Treatment decisions for our patients were made collaboratively, considering all potential risks and benefits. The patient was symptomatic and had entrapment of the lungs, as reflected by severe pain and non-expansion of the lungs upon therapeutic thoracentesis, which warranted further interventions to resolve the loculations. She was at high risk of intraoperative and postoperative complications, including delayed recovery, due to a history of coronary artery disease, low body mass index, and low albumin. She expressed reluctance to undergo surgery and showed a willingness to pursue fibrinolytics with a small-sized chest tube. Overall, the patient tolerated the procedure very well and was able to finish a three-day course with desired outcomes without significant pain.

## Conclusions

This case highlights the potential utilization of intrapleural fibrinolytics through a small-bore chest tube for loculated RPE to resolve lung entrapment, which has not been reported much in medical literature before. Intrapleural fibrinolytics in our patient were well tolerated without any significant complications and resulted in significant clinical and radiographic improvement. Further studies comparing the safety and efficacy of intrapleural fibrinolytics and surgery in RPE patients would be warranted.

## References

[1] Shaw M, Collins BF, Ho LA, Raghu G (2015). Rheumatoid arthritis-associated lung disease. Eur Respir Rev.

[2] Balbir-Gurman A, Yigla M, Nahir AM, Braun-Moscovici Y (2006). Rheumatoid pleural effusion. Semin Arthritis Rheum.

[3] Avnon LS, Abu-Shakra M, Flusser D, Heimer D, Sion-Vardy N (2007). Pleural effusion associated with rheumatoid arthritis: what cell predominance to anticipate?. Rheumatol Int.

[4] Komarla A, Yu GH, Shahane A (2015). Pleural effusion, pneumothorax, and lung entrapment in rheumatoid arthritis. J Clin Rheumatol.

[5] Bedawi EO, Stavroulias D, Hedley E (2023). Early video-assisted thoracoscopic surgery or intrapleural enzyme therapy in pleural infection: a feasibility randomized controlled trial. The third multicenter intrapleural sepsis trial-Mist-3. Am J Respir Crit Care Med.

[6] Kumar S, Akta H, Fahmawi Y, Clukies M, Lee JY (2024). Loculated rheumatoid effusions treated with alteplase-dornase. Am J Respir Crit Care Med.

[7] Chansakul T, Dellaripa PF, Doyle TJ, Madan R (2015). Intra-thoracic rheumatoid arthritis: imaging spectrum of typical findings and treatment related complications. Eur J Radiol.

[8] Yokosuka T, Suda A, Sugisaki M (2013). Rheumatoid pleural effusion presenting as pseudochylothorax in a patient without previous diagnosis of rheumatoid arthritis. Respir Med Case Rep.

[9] Faurschou P, Francis D, Faarup P (1985). Thoracoscopic, histological, and clinical findings in nine case of rheumatoid pleural effusion. Thorax.

[10] Russell ML, Gladman DD, Mintz S (1986). Rheumatoid pleural effusion: lack of response to intrapleural corticosteroid. J Rheumatol.

[11] Yigla M, Simsolo C, Goralnik L, Balabir-German A, Nahir AM (2002). The problem of empyematous pleural effusion in rheumatoid arthritis: report of two cases and review of the literature. Clin Rheumatol.

[12] Sahn SA (1988). State of the art. The pleura. Am Rev Respir Dis.

[13] Wilshire CL, Jackson AS, Vallières E (2023). Effect of intrapleural fibrinolytic therapy vs surgery for complicated pleural infections: a randomized clinical trial. JAMA Netw Open.

[14] Shirota C, Uchida H (2015). Initial treatment of septated parapneumonic empyema with drainage plus fibrinolytic agents is equally effective as video-assisted thoracoscopic surgery, and is suitable as first-line therapy. Transl Pediatr.

[15] Haag AL, Villgran VD, DiSilvio BE (2022). Rheumatoid lung treated with lytic therapy. Am J Respir Crit Care Med.

